# Supplementary material for: Experts contributions to the development of a non-sugar sweeteners warning label for Brazilian food products
Source: PLoS One. 2025 Sep 3;20(9):e0331302. doi: 10.1371/journal.pone.0331302 (PMC12407391; doi:10.1371/journal.pone.0331302)
Supplement: S1 File — Facilitation script used in expert panel sessions. (PDF) [file pone.0331302.s001.pdf]

Evaluation of the effectiveness of labeling models to identify non-sugar sweeteners presence in packaged foods and beverages in Brazil

Non-sugar sweeteners warning proposals

Panel of experts mediation guide

Location: Google Meet platform
Registration: Audio and video recording
Expected duration: 120 minutes

Activities	Stimulus/Content of speech
**Presentation and agreement | 15 min**	
Presentation of the objectives of the meeting and the health risks of the presence of non-sugar sweeteners (NSS) in foods and drinks.	**Stimulus:** Slide with the title of the panel. After all the experts join the virtual meeting, the slide is removed and the mediators open the cameras.<br>Mediators presentation and greet the experts<br>Explanation to participants about the term NSS, the health risks of the presence of this additive in food and drinks and the objectives of the Panel:<br>● Discuss, judge and make a technical decision of nutritional and design information about NSS warning label for packaged foods and beverages in Brazil, within the specific attributes of readability, visibility, attention, perception of healthiness and health risk.<br>● Subsidize the subsequent validation of models with the Brazilian population, through qualitative and quantitative methodologies.
**Expert presentation**	Participants are invited to briefly introduce themselves (name and affiliated institution).<br>After the presentations, the mediators explain the activities and procedures of the sessions.

OBSERVATION

- Send in advance a document with experts informations (name, affiliation and mini resume)

**Agreements**: sessions and activities timing	Mediators explanation:<br>● 3 sessions will be held, the purpose of which is to reach a consensus on how a warning about the presence of NSS should be presented in packaging foods.<br>● The result of each session will subsidize the next one. It is expected the experts reach a consensus or agree with their choices.<br>● For each session, a link (Google Forms) will be sent in the chat to individual answers. To respond to the form, experts must close the camera and microphone<br>● After sending the responses, a discussion will be held, with the anonymously Google forms responses shared. The objective at this moment is to listen to experts' opinions and identify the convergence of views.<br>● It should be noted that the warning proposals are aligned with Brazilian and Mercosur regulatory aspects in force and/or under discussion.<br>● 1h30min is the expected duration time for the Panel, which may vary depending on the discussions in each session.<br>After the explanation, participants are asked if they have any questions and then session 1 begins.

GENERAL NOTE

a. The on-screen presentations of the session summaries (Google forms) and the sending of links via chat will be led by a mediator.
b. At the end of each session, a short break may be necessary for forwarding links and summaries.
c. All experts should be encouraged to give their opinion.
d. Consensus must be encouraged, but not imposed by the mediator. A maximum of 2 options must occur as a result of the session, when consensus is not possible.
e. The mediator must promote a welcoming and friendly environment while conducting the sessions.
f. The discussion time must be monitored, trying not to exceed 30 minutes in each session.

Session 1: Evaluation of the warning message for NSS | 30 min

Indication by experts of the best message to identify the presence of NSS in food packaging.	**For the mediators':**<br>● Explain that the experts must choose the best warning message to indicate the presence of NSS in packaged food. To do this, a link will be made available in the chat

Individual activity followed by group discussion focusing on the reasons for the choices.

Guiding questions for the discussion (justifications):

- *What message about NSS makes products appear less healthy? Explain why.*
- *Which message makes the products seem more unhealthy? Explain why.*
- *Which message catches the most attention? Explain why.*

Topics based on the textual message: Perception of healthiness, perception of health risk, attention

with the messages and questions about them.

- Share the screen, showing what will be seen in the link (messages and questions). Ask if there are any questions about this activity.
- Request them to access the link in the chat and answer the questions. Remember that this step must be carried out with the camera and microphone closed. After responding, everyone must open their cameras and microphones to start the discussion.

(experts access the link and answer the questions)

- After all the responses, the discussion begins with a visual presentation of the Google Forms results.
- Experts are requested to justify their answers, starting with the most frequent answers.
- At the end of the discussion, 1 or 2 consensus messages are presented by the group, which must be in agreement to proceed to session 2.

BREAK TO ADJUST THE STIMULUS FOR THE NEXT SESSION

Stimulus: online form

Messages:

- CONTAINS NON-SUGAR SWEETENER
- ATTENTION: CONTAINS NON-SUGAR SWEETENER
- CONTAINS NON-SUGAR SWEETENER - NOT RECOMMENDED FOR CHILDREN
- ATTENTION: CONTAINS NON-SUGAR SWEETENER - NOT RECOMMENDED FOR CHILDREN
- CONTAINS NON-SUGAR SWEETENER: NOT RECOMMENDED FOR WEIGHT CONTROL
- ATTENTION: CONTAINS NON-SUGAR SWEETENER: NOT RECOMMENDED FOR WEIGHT CONTROL
- CONTAINS NON-SUGAR SWEETENER: NOT RECOMMENDED FOR CHILDREN AND FOR WEIGHT CONTROL

	● ATTENTION: CONTAINS NON-SUGAR SWEETENER: NOT RECOMMENDED FOR CHILDREN AND FOR WEIGHT CONTROL<br>Question 1: Which message makes products seem less healthy?<br>Question 2: Which message makes the products seem more harmful to health?<br>Question 3: Which message catches the most attention

Session 1 discussion scenarios:

1. **Consensus**: a trend is identified in the responses and those with a different position agree with the majority. Result: one option goes to session 2 and one link will be shared.
2. **Non-consensus:** a trend (or not) is identified in the responses, but those with a different position do not agree with the majority. The discussion continues until reaching at least 2 consensus options. Result: two options go to session 2. In this case, in session 2, two links will be provided for participant responses that will be discussed in the search for consensus.

Session 2: Assessment of label readability | 30 min

Expert indication/selection of the **best presentation** to the message(s) chosen in Block 1 in terms of **legibility and attention** to identifying the presence of non-sugar sweeteners in food and beverage packaging.

Individual activity followed by group discussion focusing on the reasons for the choices.

Guiding questions for the discussion (justifications):

- *Which manner is the* ***most readable****? **Why**?*
- *Which manner* ***catches more attention****? **Why**?*

Topics based on the typographic presentation of the textual message: Readability and attention

For the mediators':

- Explain that in this block, experts must indicate the best manner to visualize the warning message about non-sugar sweeteners (considering block 1 choice/s) in relation to **readability and attention**.
- Share the Links/s in the chat with questions and messages varying in the use of bold text. All options have open text (white) on a black background and in upper case/capital letters, in alignment with Brazilian and Mercosur regulatory aspects.
- Share the screen showing what they will see in the link/s (messages and questions).
- Request them to access the link in the chat and answer the questions. Remember that this step must be carried out with the camera and microphone closed. After responding, everyone must open their cameras and microphones to start the discussion.

(experts access the link and answer the questions)

- After all the responses, the discussion begins with a visual presentation of the Google Forms results.
- Experts are requested to justify their answers, starting with the most frequent answers.
- Ao final da discussão, present 1 or 2 group consensus options, which must be agreed upon to proceed to session 3.

BREAK TO ADJUST THE STIMULUS FOR THE NEXT SESSION

Stimulus: online form, presenting the messages and questions in pairs:

Exemplo:

- *What manner is the most readable?*

CONTAINS NON-SUGAR SWEETENER

CONTAINS NON-SUGAR SWEETENER

- *What manner catches more attention?*

CONTAINS NON-SUGAR SWEETENER

CONTAINS NON-SUGAR SWEETENER

Session 2 variations:

Black background with hollow text (white) and separator border varying in bold use:

- **CONTAINS** NON-SUGAR SWEETENER
- **ATTENTION**: CONTAINS NON-SUGAR SWEETENER

- **CONTAINS** NON-SUGAR SWEETENER - NOT RECOMMENDED FOR CHILDREN

- **ATTENTION**: CONTAINS NON-SUGAR SWEETENER - NOT RECOMMENDED FOR CHILDREN

- **CONTAINS** NON-SUGAR SWEETENER: NOT RECOMMENDED FOR CHILDREN AND FOR WEIGHT CONTROL

- **ATTENTION**: CONTAINS NON-SUGAR SWEETENER: NOT RECOMMENDED FOR CHILDREN AND FOR WEIGHT CONTROL

	Black background with hollow text (white) and separator border **all text in bold**:<br>● **CONTAINS NON-SUGAR SWEETENER**<br>● **ATTENTION: CONTAINS NON-SUGAR SWEETENER**<br>● **CONTAINS NON-SUGAR SWEETENER - NOT RECOMMENDED FOR CHILDREN**<br>● **ATTENTION: CONTAINS NON-SUGAR SWEETENER - NOT RECOMMENDED FOR CHILDREN**<br>● **ATTENTION: CONTAINS NON-SUGAR SWEETENER: NOT RECOMMENDED FOR WEIGHT CONTROL**<br>● **ATTENTION: CONTAINS NON-SUGAR SWEETENER: NOT RECOMMENDED FOR CHILDREN AND FOR WEIGHT CONTROL**

Session 2 discussion scenarios:

1. **Consensus:** a trend is identified in the responses and those with a different position agree with the majority and a consensus is reached in the session. Result: 1 option goes to 3 sessions.
2. **Non-consensus:** a tendency (or not) is identified in the responses, but those with a different position do not agree with the majority (there is no consensus in the bloc). The discussion continues until <u>at least 2 options are reached</u>. Result: 2 options go to session 3. In this case, in session 3, **2 links will be provided** to participants' responses that will be discussed in the search for consensus.

Session 3: Assessment of label visibility in the context of packaging | 30 min

Ranking by experts of the **visibility and attention** of the warning message on non-sugar sweeteners in the visual context of the packaging regarding its location on the **surface (front or back) and proximity to other regulated information** (magnifying glass seal, product name and list of ingredients/allergen warning).	● Explain that in this session, experts (considering the choice/s from session 2) must rank the visibility and attention of the NSS warning message in the visual context of the packaging, according to its location (front or back) and proximity to other regulated information (FoPNL, product name and list of ingredients/allergen warning). Highlight

Individual activity followed by group discussion focusing on the reasons for the choices.<br><br>Guiding questions for the discussion (justification):<br><br>● What is the **best surface and position** to present the message on non-sugar sweeteners considering its visibility on the package? Why?<br><br>● On which surface/position does the message **attract more attention**? Why?<br><br>Dimensions addressed: Visibility and attention, based on the location of the message, considering the other regulatory elements of the package.	that, depending on the packaging format in the images, the composition of the elements on the packaging varies, considering the flexibility given to the industry to present the regulated information (information in the images is fictitious).<br>● A link will be made available in the chat with questions and images of mockups of fictitious products, with the message/s selected in block 2 varying in terms of their position on the packaging on the front and back panels.<br>● Share the screen with the link that participants will see (messages and questions).<br>● After presenting the stimulus, ask them to access the link in the chat and answer the questions, remembering that this must be done with the camera and microphone closed and that after answering, everyone must open their cameras and microphones to start the discussion.<br><br>*(participants access the link and answer the questions. If there are 2 options resulting from block 2, 2 links will be sent)*<br><br>● After everyone has responded, the discussion begins with a visual presentation of the summary of responses (Google Forms)<br>● Ask participants to justify their answers, starting with the most common answers.<br>● At the end of the discussion, 1 or 2 consensus options are presented by the group, which must be agreed upon in order to proceed to the finalization of the Panel.<br>● With this, the mediators of this session close the discussion.<br><br>**About the stimulus: online form (Google forms) presenting:**<br><br>● Mockups varying in packaging shape (circular, vertical rectangular, horizontal rectangular)<br>● Messages vary in position on the front of the packaging: next to the magnifying glass or the product name

	● The messages vary in position on the back: next to the list of ingredients and warning about allergens.<br>● Group images will be provided, as presented in the questions, but in A4 format (pdf) for better viewing. This is because Google forms only display images in small sizes.<br><br>The questions below show the products with the message in the same location:<br><br>Example:<br><br>● What is the best face and position to present the message about Sweeteners considering its visibility on the packaging?<br><br>*[Front face: all products with messages next to the FoPNL]*<br><br>*[Front face: all products with messages next to the product name]*<br><br>*[Back face: all products with messages next to the list of ingredients and allergen warning]*<br><br>● In which location/position does the message attract the most attention?<br><br>*[Front side: all products with messages next to the magnifying glass]*<br><br>*[Front side: all products with messages next to the product name]*<br><br>*[Back side: all products with messages next to the list of ingredients and allergen warning]*

End of session | 15 min

Summary of the results	**For the mediators':**<br><br>● Mediators summarize the aspects discussed and the results/outputs generated, resuming the Panel's objectives.<br><br>● The block choices and possible consensuses are visually shown<br>● Provide a moment for participant comments<br>● Report next actions based on the contributions of the Expert Panel.<br>● Closing session with thanks to the experts.
