# Supplementary material for: Experts contributions to the development of a non-sugar sweeteners warning label for Brazilian food products
Source: PLoS One. 2025 Sep 3;20(9):e0331302. doi: 10.1371/journal.pone.0331302 (PMC12407391; doi:10.1371/journal.pone.0331302)
Supplement: S2 File — Questionnaire items and format used in the Google Form. (PDF) [file pone.0331302.s002.pdf]

Session 1

Evaluate the content of the warning message for non-sugar sweeteners

* Indicates required question

1. Your area of expertise

(Short answer)

2. Which message about non-sugar sweeteners leads to the perception that products are less healthy?*

Mark only one oval.

- CONTÉM EDULCORANTE
  (in English: CONTAINS NON-SUGAR SWEETENER)
- ATENÇÃO: CONTÉM EDULCORANTE
  (in English ATTENTION: CONTAINS NON-SUGAR SWEETENER)
- CONTÉM EDULCORANTE - NÃO RECOMENDADO PARA CRIANÇAS
  (in English CONTAINS NON-SUGAR SWEETENER - NOT RECOMMENDED FOR CHILDREN)
- ATENÇÃO: CONTÉM EDULCORANTE - NÃO RECOMENDADO PARA CRIANÇAS
  (in English ATTENTION: CONTAINS NON-SUGAR SWEETENER - NOT RECOMMENDED FOR CHILDREN)
- CONTÉM EDULCORANTE - NÃO RECOMENDADO PARA CONTROLE DE PESO
  (in English CONTAINS NON-SUGAR SWEETENER - NOT RECOMMENDED FOR WEIGHT CONTROL)
- ATENÇÃO: CONTÉM EDULCORANTE - NÃO RECOMENDADO PARA CONTROLE DE PESO
  (in English ATTENTION: CONTAINS NON-SUGAR SWEETENER: NOT RECOMMENDED FOR WEIGHT CONTROL)
- CONTÉM EDULCORANTE - NÃO RECOMENDADO PARA CRIANÇAS E PARA CONTROLE DE PESO
  (in English CONTAINS NON-SUGAR SWEETENER: NOT RECOMMENDED FOR CHILDREN AND FOR WEIGHT CONTROL)

3. Which message causes products to be perceived as more harmful to health?*

Mark only one oval.

- CONTÉM EDULCORANTE
  (in English: CONTAINS NON-SUGAR SWEETENER)
- ATENÇÃO: CONTÉM EDULCORANTE
  (in English ATTENTION: CONTAINS NON-SUGAR SWEETENER)
- CONTÉM EDULCORANTE - NÃO RECOMENDADO PARA CRIANÇAS
  (in English CONTAINS NON-SUGAR SWEETENER - NOT RECOMMENDED FOR CHILDREN)
- ATENÇÃO: CONTÉM EDULCORANTE - NÃO RECOMENDADO PARA CRIANÇAS
  (in English ATTENTION: CONTAINS NON-SUGAR SWEETENER - NOT RECOMMENDED FOR CHILDREN)
- CONTÉM EDULCORANTE - NÃO RECOMENDADO PARA CONTROLE DE PESO
  (in English CONTAINS NON-SUGAR SWEETENER - NOT RECOMMENDED FOR WEIGHT CONTROL)
- ATENÇÃO: CONTÉM EDULCORANTE - NÃO RECOMENDADO PARA CONTROLE DE PESO
  (in English ATTENTION: CONTAINS NON-SUGAR SWEETENER: NOT RECOMMENDED FOR WEIGHT CONTROL)
- CONTÉM EDULCORANTE - NÃO RECOMENDADO PARA CRIANÇAS E PARA CONTROLE DE PESO
  (in English CONTAINS NON-SUGAR SWEETENER: NOT RECOMMENDED FOR CHILDREN AND FOR WEIGHT CONTROL)

4. Which message attracts the most attention?*

Mark only one oval.

- CONTÉM EDULCORANTE
(in English: CONTAINS NON-SUGAR SWEETENER)
- ATENÇÃO: CONTÉM EDULCORANTE
(in English ATTENTION: CONTAINS NON-SUGAR SWEETENER)
- CONTÉM EDULCORANTE - NÃO RECOMENDADO PARA CRIANÇAS
(in English CONTAINS NON-SUGAR SWEETENER - NOT RECOMMENDED FOR CHILDREN)
- ATENÇÃO: CONTÉM EDULCORANTE - NÃO RECOMENDADO PARA CRIANÇAS
(in English ATTENTION: CONTAINS NON-SUGAR SWEETENER - NOT RECOMMENDED FOR CHILDREN)
- CONTÉM EDULCORANTE - NÃO RECOMENDADO PARA CONTROLE DE PESO
(in English CONTAINS NON-SUGAR SWEETENER - NOT RECOMMENDED FOR WEIGHT CONTROL)
- ATENÇÃO: CONTÉM EDULCORANTE - NÃO RECOMENDADO PARA CONTROLE DE PESO
(in English ATTENTION: CONTAINS NON-SUGAR SWEETENER: NOT RECOMMENDED FOR WEIGHT CONTROL)
- CONTÉM EDULCORANTE - NÃO RECOMENDADO PARA CRIANÇAS E PARA CONTROLE DE PESO
(in English CONTAINS NON-SUGAR SWEETENER: NOT RECOMMENDED FOR CHILDREN AND FOR WEIGHT CONTROL)

Thank you for your responses in this session.

Session 2

Label readability assessment

* Indicates required question

1. Your area of expertise

(Short answer)

2. Which format for presenting the message about non-sugar sweeteners is the most legible?*

Consider that, according to legislation, font size and typeface will be standardized regardless of word count (e.g., 6 pt font, Helvetica).

Mark only one oval.

- Entire sentence in bold
- Only the word "attention" in bold

3. Which message presentation format attracts the most attention?*

Consider that, according to legislation, font size and typeface will be standardized regardless of word count (e.g., 6 pt font, Helvetica).

Mark only one oval.

- Entire sentence in bold
- Only the word "attention" in bold

Thank you for your responses in this session.

Session 3

Evaluation of label visibility within packaging context

* Indicates required question

1. Your area of expertise

(Short answer)

The attached images show **variations in the placement** of the warning message about non-sugar sweeteners on the front or back of the packaging, as well as its proximity to other regulatory information (e.g., next to the FoPNL, after the list of ingredients, next to the sales denomination). Please **rank** the options below according to the **visibility** and **attention** attracted by the warning message within the visual context of the packaging.

2. Rank the options below according to the visibility of the non-sugar sweetener warning message within the visual context of the packaging.

Mark only one oval per row.

	Next to the FoPN	After the list of ingredients	Next to the sales denomination
**Fist option**			
**Second option**			
**Third option**			

3. Rank the options below according to the attention drawn by the non-sugar sweetener warning message within the visual context of the packaging.

Mark only one oval per row.

	Next to the FoPN	After the list of ingredients	Next to the sales denomination
**Fist option**			
**Second option**			
**Third option**			

Thank you for your responses in this session.
