# Supplementary material for: Experts contributions to the development of a non-sugar sweeteners warning label for Brazilian food products
Source: PLoS One. 2025 Sep 3;20(9):e0331302. doi: 10.1371/journal.pone.0331302 (PMC12407391; doi:10.1371/journal.pone.0331302)
Supplement: S3 File — Codebook used for the qualitative content analysis of experts’ responses. (PDF) [file pone.0331302.s003.pdf]

Expert panel codebook

code/subcode	short name	short description	detailed description	inclusion criteria	exclusion criteria	typical example	atypical example	close but no
**Attention**	Attention	Statements related to attention	Expert statements regarding the use of the word "attention" signal and how much the labeling models for sweeteners presented attract attention when seen.					
**The attribute of attracting attention**	attract attention	Statements related to attracting attention	Experts' statements regarding the FoP NSS warning labels attract attention when they are seen	Statements referring to the attention provided by the proposed labeling models for NSS.	Statements that do not mention the attention provided by the proposed labeling models for NSS.	The criterion was to draw people's attention, right? To show that something could be, present a risk. I think the word 'ATTENTION' makes some difference. I've heard this from some people and particularly for me too, if I see an 'ATTENTION', that already sets off a red light in my head.	I was talking about mobility, that in fact our gaze is tactile and mobility is in fact attractive, but in the context, where the other information is already in bold, ideally then, everything would be in bold.	Yes, just 'ATTENTION' [in bold], because there is a question of mobility of the gaze, right. Our gaze is tactile. So, when we have mobility, the tendency to be attracted by the mobility of the gaze itself is greater. So, 'ATTENTION' as it is called in bold and then you continue reading. That was the reason.

**Use of the word attention as a sign**	Term attentio n	Statements related to the term "attention"	Experts' statements regarding the use of the term "attention" in the FoP NSS warning labels	Statements mentioning the use of the term "attention" in the messages of the proposed labeling models for NSS.	Statements that do not mention the term "attention" present in the messages of the proposed labeling models for NSS.	First of all, what I wanted to talk about is the word 'attention' at the beginning of the sentence. I think it puts you in a position, prepares you, leaves you ready.	I reinforce this perception with regard to the term 'ATTENTION' and the short message	Yeah, but I stopped at the sweetener, I marked 'WARNING: CONTAINS NON-SUGAR SWEETENER
**Attribute s about FoP NSS warning labels**	Labelin g of NSS	Statements related to model attributes on labels	Experts' statements regarding the attributes present within the labeling models (message, types of presentation, alert size, etc.) proposed to indicate the presence of NSS and on packaged foods and beverages.	Statements that address the size of the message/amount of information that will be present within the proposed sweetener labeling models, which may include the arrangement of the information considering other elements of the labels.	Statements that do not address the size of the message to indicate the presence of sweeteners in packaged foods and beverages	"When we use front labeling, we use front labeling in a very direct way, so the more letters that appear, the more confusing I believe it will be for the population"	"In terms of labeling, the simpler the label, the better the message gets across. So I think 'CONTAINS SWEETENER' would be ideal."	"It's front labeling that you're working on, so there's an issue, the larger text will be worked on in a smaller size and it could harm attention in that sense, because I think that's an issue that I wanted to raise as well."
**Inferenc e about consum ers opinions**	Inferen ce about opinion	Statements about the possible opinion and/or interpretation of consumers	Experts' statements with inferences about the possible opinion and/or interpretation of consumers regarding the labeling models for NSS in packaged foods and beverages.	Statements that were directly or indirectly related to the possible opinion and/or interpretation of consumers	Statements that were not related, directly or indirectly, to the possible opinion and/or interpretation of consumers	"I think the longer the sentence, the less likely people are to read it, so depending on the packaging, depending on the situation, people might get a little lazy, "too much information, I'm not going to read it", I think that's a point. A more direct	"If I put something that I think the consumer will find less healthy, then I go back to that huge sentence and I'll also put comma and teenager comma and pregnant comma whoever is breastfeeding comma, and whoever doesn't like that	"The criterion was to draw people's attention, right? To show that something could be, present a risk. I think the word 'ATTENTION' makes some difference"

						message, many times, many times not, we don't know, can have more effect, right, thinking"	aftertaste, sometimes sugar is better than sweetener, you know?"	
**No recommendation to consume NSS**	No recommendation of NSS	Statements regarding the non-recommended consumption of NSS	Experts' statements with arguments regarding not recommending the consumption of NSS for different public, contemplating scientific evidence arguments	Statements that address the non-recommendation of consumption of NSS by different audiences, also including scores on the strength of the scientific evidence used as a basis for the claim	Statements that mention the term sweetener, but do not relate it to its non-recommended consumption	"And so, I think a little about the criteria for choosing which sweetener is not recommended. Of course, we know that there is the issue of children, from the point of view of public health and additives being potentially more toxic to them, the issue of weight control according to WHO recommendations, but I think that is not all."	"I was very undecided, but what made me select this option was because I feel that when we specify the target audience, this could exclude me from the audience for whom it is harmful. So, I may have the idea that if this is not suitable for children and for those who are in weight control, then perhaps it is not suitable for me, who is not a child and does not have weight control."	"The other aspect is in relation to continuity, 'NOT RECOMMENDED FOR CHILDREN', this already evidently places the child as not recommended and again, reinforces, draws attention"

**Perception of healthiness and/or health risk**	Healthiness and health risk	Statements about the perception of healthiness and/or health risk	Experts' statements regarding their perception of healthiness and/or health risk in relation to the FoP NSS warning labels.	Statements that position labeling models for sweeteners as healthier or more harmful to health, potentially making inferences about the opinion of consumers.	Statements that address healthiness and/or health risks, without relating to the proposed labeling models for NSS.	"It seemed to me that when we contraindicate its use for children and for weight control, people will not know what it is, but will have a negative bias towards the product precisely because there is no contraindication"	"ATTENTION: CONTAINS NON-SUGAR SWEETENER" in my interpretation is not ideal to say that it is more harmful to health or I don't remember which one is the previous one, it is not ideal. But it is ideal to inform the population what the food contains, so I think they are slightly different concepts, right?"	"If I'm not concerned about weight control, and I'm not even a child, I would be covered, right? This product would be recommended for me, sorry, and it isn't, so I would also like to know, as a healthy adult, I would like to know that this product is not recommended for me."
**Expert's personal preference/opinion**	Expert preference/opinion	Statements that address the experts' preferences and opinions	Experts' statements indicating their personal and/or professional preference/opinion on the FoP NSS warning labels, considering possible mentions or disagreements regarding the questions asked in the panel dynamics.	Statements that address the expert's personal and/or professional preference regarding the proposed labeling models for NSS	Statements that deal with the specialist's personal and/or professional preference and are not related to the proposed labeling models for sweeteners	"What I missed when I looked at the block of questions there was “what is your opinion about this?” Because if it were my opinion, it would just be ‘CONTAINS SWEETENER’"	"So, I wanted to make it very clear that I did X, as it seems, what it seems is this, but it does not mean adherence to this solution"	"I also agree with the issue of the long sentence, especially in the context of a package. If you have a lot of text, it is obviously not what will attract the most attention."
