# Supplementary material for: Experts contributions to the development of a non-sugar sweeteners warning label for Brazilian food products
Source: PLoS One. 2025 Sep 3;20(9):e0331302. doi: 10.1371/journal.pone.0331302 (PMC12407391; doi:10.1371/journal.pone.0331302)
Supplement: Attachment — Submitted filename: Comments-PONE-D-25-27597.pdf [file pone.0331302.s004.pdf]

PLOS ONE

# Experts contributions to the development of a non-sugar sweeteners warning label for Brazilian food products

--Manuscript Draft--

| | |
|---|---|
| Manuscript Number: | PONE-D-25-27597 |
| Article Type: | Research Article |
| Full Title: | Experts contributions to the development of a non-sugar sweeteners warning label for Brazilian food products |
| Short Title: | Experts contributions to non-sugar sweeteners warning label |
| Corresponding Author: | Mariana Ribeiro<br>University of Sao Paulo: Universidade de Sao Paulo<br>São Paulo, São Paulo BRAZIL |
| Keywords: | Sweeteners; Food Labeling; Public Health Policies; Ultraprocessed Foods |
| Abstract: | The consumption of non-sugar sweeteners (NSS) has recently been associated with potential health risks, including glucose intolerance, alterations in the intestinal microbiota which lead to metabolic abnormalities including pancreatic endocrine dysfunction, aggravation of kidney disease, increased risk of cancer and adverse cardiovascular outcomes. In Brazil, the presence of NSS in ultra-processed food products (UPFP) is only reported in ingredient lists, which Brazilian consumers struggle to locate and understand. On the other hand, countries such as Argentina and Mexico have implemented front-of-pack nutrition warning labels (FoPNWL) specifically for NSS in packaged foods and beverages. Thus, this study aimed to evaluate expert opinion on the design attributes of NSS warning labels that have been proposed to inform consumers about the presence of these sweeteners in packaged foods and beverages sold in Brazil. For this purpose, a panel of experts was conducted to discuss, judge and decide on the technical aspects of readability, visibility, attention, perceived healthiness and health risk of the proposed warning labels for NSS in packaged foods and beverages in Brazil. The experts identified two most suitable NSS warning messages: "attention: contains sugar-free sweetener - not recommended for children and for weight control" and "attention: contains non-sugar sweetener". |
| Order of Authors: | Mariana Ribeiro |
| | Priscila de Morais Sato |
| | Carlos Felipe Urquizar Rojas |
| | Carla Galvão Spinillo |
| | Laís Amaral Mais |
| | Camila Aparecida Borges |
| | Ana Paula Bortoletto Martins |
| Additional Information: | |
| Question | Response |
| **Financial Disclosure**<br><br>Enter a financial disclosure statement that describes the sources of funding for the work included in this submission. Review the submission guidelines for detailed requirements. View published research articles from *PLOS ONE* for specific examples. | The author(s) received no specific funding for this work. |

Powered by Editorial Manager® and ProduXion Manager® from Aries Systems Corporation

| | |
|---|---|
| This statement is required for submission and **will appear in the published article** if the submission is accepted. Please make sure it is accurate.<br><br>**Funded studies**<br>Enter a statement with the following details:<br>• Initials of the authors who received each award<br>• Grant numbers awarded to each author<br>• The full name of each funder<br>• URL of each funder website<br>• Did the sponsors or funders play any role in the study design, data collection and analysis, decision to publish, or preparation of the manuscript?<br><br>Did you receive funding for this work? | |
| **Competing Interests**<br><br>Use the instructions below to enter a competing interest statement for this submission. On behalf of all authors, disclose any competing interests that could be perceived to bias this work—acknowledging all financial support and any other relevant financial or non-financial competing interests.<br><br>This statement is required for submission and **will appear in the published article** if the submission is accepted. Please make sure it is accurate and that any funding sources listed in your Funding Information later in the submission form are also declared in your Financial Disclosure statement.<br><br>View published research articles from *PLOS ONE* for specific examples. | The authors have declared that no competing interests exist. |

| | |
|---|---|
| **NO authors have competing interests**<br><br>Enter: *The authors have declared that no competing interests exist.*<br><br>**Authors with competing interests**<br><br>Enter competing interest details beginning with this statement:<br><br>*I have read the journal's policy and the authors of this manuscript have the following competing interests: [insert competing interests here]*<br><br>* typeset | |
| **Ethics Statement**<br><br>Enter an ethics statement for this submission. This statement is required if the study involved:<br><br>• Human participants<br>• Human specimens or tissue<br>• Vertebrate animals or cephalopods<br>• Vertebrate embryos or tissues<br>• Field research<br><br>Write "N/A" if the submission does not require an ethics statement.<br><br>General guidance is provided below. Consult the submission guidelines for detailed instructions. **Make sure that all information entered here is included in the Methods section of the manuscript.** | Human participants.<br>This study was approved by the Ethics Committee of the University of São Paulo (protocol n. 69441423.2.0000.5421).<br>The panel of experts was conducted after participants had given written informed consent. |

Powered by Editorial Manager® and ProduXion Manager® from Aries Systems Corporation

| | |
|---|---|
| **Format for specific study types**<br><br>**Human Subject Research (involving human participants and/or tissue)**<br>• Give the name of the institutional review board or ethics committee that approved the study<br>• Include the approval number and/or a statement indicating approval of this research<br>• Indicate the form of consent obtained (written/oral) or the reason that consent was not obtained (e.g. the data were analyzed anonymously)<br><br>**Animal Research (involving vertebrate animals, embryos or tissues)**<br>• Provide the name of the Institutional Animal Care and Use Committee (IACUC) or other relevant ethics board that reviewed the study protocol, and indicate whether they approved this research or granted a formal waiver of ethical approval<br>• Include an approval number if one was obtained<br>• If the study involved *non-human primates*, add *additional details* about animal welfare and steps taken to ameliorate suffering<br>• If anesthesia, euthanasia, or any kind of animal sacrifice is part of the study, include briefly which substances and/or methods were applied<br><br>**Field Research**<br><br>Include the following details if this study involves the collection of plant, animal, or other materials from a natural setting:<br>• Field permit number<br>• Name of the institution or relevant body that granted permission | |
| **Data Availability**<br><br>Authors are required to make all data underlying the findings described fully available, without restriction, and from the time of publication. PLOS allows rare exceptions to address legal and ethical concerns. See the PLOS Data Policy and FAQ for detailed information. | Yes - all data are fully available without restriction |

| | |
|---|---|
| A Data Availability Statement describing where the data can be found is required at submission. Your answers to this question constitute the Data Availability Statement and **will be published in the article**, if accepted.<br><br>**Important:** Stating 'data available on request from the author' is not sufficient. If your data are only available upon request, select 'No' for the first question and explain your exceptional situation in the text box.<br><br>Do the authors confirm that all data underlying the findings described in their manuscript are fully available without restriction? | |
| **Describe where the data may be found in full sentences. If you are copying our sample text, replace any instances of XXX with the appropriate details.**<br><br>• If the data are **held or will be held in a public repository**, include URLs, accession numbers or DOIs. If this information will only be available after acceptance, indicate this by ticking the box below. For example: *All XXX files are available from the XXX database (accession number(s) XXX, XXX.).*<br>• If the data are all contained **within the manuscript and/or Supporting Information files**, enter the following: *All relevant data are within the manuscript and its Supporting Information files.*<br>• If neither of these applies but you are able to provide **details of access elsewhere**, with or without limitations, please do so. For example:<br><br>*Data cannot be shared publicly because of [XXX]. Data are available from the XXX Institutional Data Access / Ethics Committee (contact via XXX) for researchers who meet the criteria for access to confidential data.*<br><br>*The data underlying the results presented in the study are available from (include the name of the third party* | All relevant data are within the manuscript and its Supporting Information files |

| | |
|---|---|
| *and contact information or URL).*<br>• This text is appropriate if the data are owned by a third party and authors do not have permission to share the data.<br><br>* typeset | |
| Additional data availability information: | |

Manuscript
Click here to access/download;Manuscript;finalversion_Experts

# Experts contributions to the development of a non-sugar sweeteners warning label for Brazilian food products

Mariana Ribeiro*[1,2,3] , Priscila de Morais Sato[4¶], Carlos Felipe Urquizar Rojas[5,6¶], Carla Galvão Spinillo[6¶], Laís Amaral Mais[2&], Camila Aparecida Borges[3,7&], Ana Paula Bortoletto Martins[1,3]

1. School of Public Health, University of São Paulo (FSP/USP), São Paulo, São Paulo, Brasil.

2. Institute of Consumers Defense (Idec), São Paulo, São Paulo, Brasil.

3. Center for Epidemiological Research in Nutrition and Health, University of São Paulo (Nupens/USP), São Paulo, São Paulo, Brasil.

4. School of Nutrition, Federal University of Bahia (ENUFBA), Salvador, Bahia, Brasil.

5. Design Department of the University of the Joinville Region (UNIVILLE), Joinville, Santa Catarina, Brasil.

6. Design Department of the Federal University of Paraná (UFPR), Curitiba, Paraná, Brasil.

7. Luiz de Queiroz College of Agriculture of University of São Paulo (ESALQ - USP), Piracicaba, São Paulo, Brasil.

*Corresponding Author

E-mail: mariana.ribeiro@idec.org.br (MR)

¶ These authors contributed equally to this work.

& These authors also contributed equally to this work.

# Abstract

The consumption of non-sugar sweeteners (NSS) has recently been associated with potential health risks, including glucose intolerance, alterations in the intestinal microbiota which lead to metabolic abnormalities including pancreatic endocrine dysfunction, aggravation of kidney disease, increased risk of cancer and adverse cardiovascular outcomes. In Brazil, the presence of NSS in ultra-processed food products (UPFP) is only reported in ingredient lists, which Brazilian consumers struggle to locate and understand. On the other hand, countries such as Argentina and Mexico have implemented front-of-pack nutrition warning labels (FoPNWL) specifically for NSS in packaged foods and beverages. Thus, this study aimed to evaluate expert opinion on the design attributes of NSS warning labels that have been proposed to inform consumers about the presence of these sweeteners in packaged foods and beverages sold in Brazil. For this purpose, a panel of experts was conducted to discuss, judge and decide on the technical aspects of readability, visibility, attention, perceived healthiness and health risk of the proposed warning labels for NSS in packaged foods and beverages in Brazil. The experts identified two most suitable NSS

warning messages: “attention: contains sugar-free sweetener - not recommended for children and for weight control” and “attention: contains non-sugar sweetener”.

# Introduction

Non-sugar sweeteners (NSS) are a functional class of food additives (FA) defined in Brazil as substances other than sugars that provide a sweet taste to foods and beverages(1). They were developed as non-caloric substitutes for sugars (2), and are currently in widespread use, mainly as a component of ultra-processed food products (UPFP), either combined with added sugars or not (3).

UPFP is a food category defined by the Nova classification, a system that categorizes food groups regarding their extent and purpose of industrial processing(4). The presence of a NSS is considered a marker to identify UPFP, as they belong to the class of food additives with cosmetic functions. Those types of food additives are used to make the final product palatable or hyper-palatable, and include substances such as flavorings, flavor enhancers, colorings, emulsifiers, emulsifying salts, thickeners, antifoaming, bulking, carbonating, foaming, gelling and glazing agents, besides NSS(3). Concurrently, consumption of UPFP has increased worldwide over the last decade, and a consolidated body of scientific evidence associates these products with negative health outcomes(5–11). These findings support the recommendations of the Brazilian Dietary Guidelines, which advise avoiding the consumption of UPFP and favoring fresh or minimally processed foods, as well as culinary preparations made from these foods(12).

Recent scientific evidence has associated the consumption of NSS with potential health risks(13), such as glucose intolerance and alterations in the intestinal

microbiota, which can lead to metabolic abnormalities (including pancreatic endocrine dysfunction(14–16). NSS intake has also been associated with the worsening of kidney disease (17), as well as an increased risk of cancer (18) and adverse cardiovascular outcomes (19). Specifically, the consumption of aspartame, sucralose, saccharin and acesulfame potassium are associated with alterations in normal physiological processes involved in the absorption and metabolism of nutrients, contributing to increased abdominal adiposity and body mass index (BMI) in adults and older individuals (20–22).

Following a systematic review and meta-analysis of the use of  NSS (13), the World Health Organization (WHO) has published guidelines regarding the consumption of NSS in 2023. The document presents a conditional recommendation of the WHO advising against the use of NSS for weight control or for reducing the risk of developing non-communicable diseases (NCD)(23).

Given that most foods and beverages are purchased in packaged form, labeling plays a crucial role in informing consumers about the presence of NSS. In Brazil, the presence of NSS in UPFP is currently disclosed only in the ingredients list – a section that is often difficult for consumers to locate and interpret  (24). Based on a public health and consumer rights approach, Argentina and Mexico have enacted legislation mandating front-of-package nutrition warning labels (FoPNWL) for the presence of NSS in packaged foods and beverages (25,26).

Warning labels are an effective strategy to empower consumers by enhancing their understanding of the composition of packaged food products and supporting healthier food choices (27–29), by particularly by mitigating the influence of health and nutrition claims (30). For a warning to effectively communicate risk, it must include graphic elements that enhance visibility and comprehension (31). The visual

environment of a product package includes branding, marketing elements and nutrition claims(32),  so warning labels must clearly stand out by using specific design features such as shape, size, typography, and layout to ensure visibility, readability, and comprehension(33). Color also plays a critical role, with black being the most used color for regulatory warnings in nutrition labeling (34–37).

An example of a cautionary label regarding the presence of NSS is found in Argentina and Mexico, where it is accompanied by a message advising against consumption by children. This warning is displayed within a black rectangle with white lettering, making it stand out compared to other packaging elements. The literature further suggests that such messages should begin with a signal word, such as "Attention" or "Caution," followed by a description of the risk and a recommendation to avoid the product. Chile was one the first countries in the world to approve and implement a front-of-package nutrition warning labeling (FoPNL) for nutrients in excess (e.g., High in sodium) (38). The Chilean Food Labeling and Advertising Law represents a significant advancement in this area; however, one limitation is the absence of a warning to indicate the presence of NSS. Following its implementation, the country experienced widespread UPFP reformulation, characterized by the total or partial replacement of added sugars with NSS, to keep the sweet taste of products. As a result, an increasing number of UPFP became available without the “high in sugar” FoPNL (39,40).

Given the growing regarding NSS consumption, the implementation of the Brazilian FoPNL makes identifying NSS in UPFP particularly relevant. In 2020, Brazil approved a new nutrition labelling regulation, which mandates a FoPNL featuring a magnifying glass icon and the expression “high in” for three nutrients of concern: added sugar, saturated fat and sodium. The regulation follows a phased

implementation timeline based on product categories. It began in October 2022, for news products, and will be fully in effect in October 2025, for non-alcoholic beverages in returnable packaging (41,42). Similar to Chile, the Brazilian FoPNL does not include a warning for NSS. Based on the Chilean experience, it is expected that reformulation through the total or partial replacement of added sugars for NSS will also occur in the Brazilian market.

To uphold the right to information - and in the light of WHO guidelines and the NSS labeling models adopted in Argentina and Mexico - it is essential to develop a specific NSS labelling strategy for the Brazilian population. Such strategy should ensure that information is clear, visible and easy-to-interpret, enabling consumers to make more informed and potentially healthier food choices. In this context, the present study aimed to evaluate experts' opinions on specific attributes of proposed NSS warning labels designed to inform consumers about the presence of NSS in packaged foods and beverages sold in Brazil.

# Materials and methods

## Study design

A qualitative study was conducted using a panel of experts. This approach is defined as a data collection method for exploratory research, aimed at sharing informed opinions based on current evidence, through discussions and debates among specialists. Its purpose is to support argumentation on emerging or unconsolidated topics, by reaching consensus or identifying key priorities and potential pathways for advancement (43,44). In this study, the experts panel aimed to discuss, evaluate and decide - at a technical level – on aspects suchs as legibility, visibility,

attention and perception of healthiness and health risk associated with warning labels proposals to inform consumers about the presence of NSS in packaged foods and beverages sold in Brazil.

The warning proposals were developed by the Institute for Consumers' Defense (Idec) and the Laboratory of Information Design Systems at the Federal University of Paraná (LabDSI/UFPR). These proposals were based on the WHO guidelines on NSS use, the warning labels approved by Argentina and Mexico, and discussions within the Southern Common Market – MERCOSUR, as well as and the existing warning label literature. Design elements included a signal word, message typographic emphasis and a black box format. The researchers involved in this study were nutritionists and information designers affiliated with public universities or a non-governmental organization (NGO) in Brazil. This study represents the first phase of a broader investigation and aims to support the validation of these models among the Brazilian population.

## Sampling

Expert selection considered the following criteria: academic affiliation, regional representation across Brazil and expertise in food and beverage labeling, NSS and/or food advertising. A purposive sample strategy was employed and invitation were sent via email exclusively to Brazilian experts. The exclusive use of the Portuguese language in the proposed labeling models was a consideration, given the potential for variation in interpretation when translated into other languages. The panel included experts from diverse fields such as social communication, information design, law, pharmacy, medicine, nutrition and psychology.

## Material and Procedures

The panel of experts was conducted online on October 3, 2023, from 4pm to 6pm, via the Google Meet platform. The session was moderated by four researchers involved in the study. An online questionnaire, administered via Google Forms during the meeting, was used to collect individual responses. Experts provided consent for your participation by completing the consent form previously sent. The entire meeting was recorded and transcribed for subsequent content analysis.

The two-hour meeting was divided into three sessions (30 minutes each), in addition to an initial moment (15 minutes) to greet the experts' and firm the agreements, and a final moment (15 minutes) to close the session. The sessions were interconnected, with the outcomes of one influencing the next. To conduct the panel, a mediation guide (Supplementary S.1) was developed, covering the following topics: evaluation of the warning message for NSS, evaluation of label readability and evaluation of label visibility in the context of packaging.

Different Google Forms (Supplementary S.1) were developed and shared through the meeting chat during each session. Experts submitted their responses anonymously prior to group discussions. Since the session involved the evaluation of visual elements (e.g., label mockups), participants were required to use a laptop or desktop computer. In each session, a Google Form link was shared with the participants and, after completing the questionnaire individually, the experts presented and justified their choices in a group debate. Transition to the next session required agreement on the previous topic, as each step built upon the previous findings. The first session aimed to evaluate the content of the warning message for sweeteners,

the second to evaluate the readability of the chosen message within a warning model, and the third to evaluate the visibility of the message within a warning model in the context of product mockup packaging.

The first session (evaluation of the NSS warning message) aimed to identify the most appropriate sentence for the NSS warning label, based on the expert's opinions (Fig 1).

**Fig 1. Sentences to be evaluated by the experts based on the best message to indicate the presence of NSS in food products**.

Based on the first session's discussion, the second session addressed label readability. Experts assessed the visual presentation of the NSS warning label, with an emphasis on readability and attention capture. Participants were shown different label formats and asked to indicate which version was more readable and more attention-grabbing (Fig 2).

**Fig 2. Examples of NSS warning labels to evaluate the readability and attention by the experts.**
Note: The texts in Portuguese language are the same as those shown in Fig 1.

The third and last session (assessment of label visibility in the context of packaging) aimed to analyze NSS warning labels on food and beverage mockups, positioned in different locations. Two previous studies were considered to select the food categories of the mockups: Brazilian Household Budget Survey (*Pesquisa de Orçamentos Familiares* - POF) from 2008-2009 and from 2017-2018(45), and a Chilean study, which brings the food categories whose use of NSS increased after the implementation of the Food Labeling and Advertising Law (40). Four product categories were chosen: ready to drink fruit juice, sandwich biscuit, dairy drink and

fermented milk. Labels were shown in the following positions: (A) main panel near the FoPNL, (B) main panel near the product name, and (C) back panel near the ingredient list and allergen warnings (Fig 3).

**Fig 3. Food and beverage mockups with different positions of NSS warning label** (A) main panel and near the FoPNL. (B) main panel and near to sales denomination. (C) back panel and near to list of ingredients and allergen warning.

In the final form, experts were asked to individually rank each label configuration based on visibility and attention. The session concluded with a final group discussion, informed by the ranking results.

# Data analysis

## Questionnaire analysis

Responses submitted individually via the questionnaires were organized by session and analyzed to identify the results corresponding to each objective. Data related to the individual questionnaires will be indicated below.

## Content analysis

The entire panel was recorded and transcribed verbatim. A thematic content analysis approach was used to interpret the experts' discussions (46). The first author (MR) read the transcript thoroughly, made notes, and identified salient themes. An exploratory analysis was conducted to identify pre-codes (*a priori* and emerging codes), that reflected key aspects of the discussions. These informed the development of the codebook (Supplementary S.2). Considering the mediation guide and the exploratory analysis, MR proposed the list of codes, and their definitions, which were discussed with the co-authors until consensus was reached. The finalized codebook

included: code; subcode; short name; short and detailed description; inclusion and exclusion criteria; typical and atypical examples; and a “close but no” example to clarify code bounderies. Coding of expert statements was conducted using NVivo 14 software (Lumivero).

# Results

The panel was formed by 10 experts from different knowledge areas, such as: nutrition (n = 4), social communication (n = 1); information design (n = 1), law (n = 1), pharmacy (n = 1), medicine (n = 1) and psychology (n = 1). Participants were from different Brazilian macroregions: Southeast (n = 5), South (n = 2), Northeast (n = 1) and Central-West (n = 1).

Based on the results of the individual questionnaire and the collective discussion, some changes in expert opinion were observed. The predefined questions in the Google Forms focused on technical attributes of design (readability, visibility and attention) and health (perception of healthiness and health risk). In contrast, the collective discussion introduced and prioritized emerging aspects, such as inferences about Brazilian consumers’ opinions and the expert's personal preference.

Regarding the NSS warning label message, "ATTENTION: CONTAINS NON-SUGAR SWEETENER: NOT RECOMMENDED FOR CHILDREN AND FOR WEIGHT CONTROL" (“*ATENÇÃO: CONTÉM EDULCORANTE - NÃO RECOMENDADO PARA CRIANÇAS E PARA CONTROLE DE PESO*”, in Portuguese) was selected in the individual questionnaire as the one that makes products seem less healthy (n = 4), makes products seem more harmful to health (n = 6) and attracts the most attention (n = 6). The second most selected message was “ATTENTION: CONTAINS NON-SUGAR SWEETENER” (*ATENÇÃO: CONTÉM EDULCORANTE*, in Portuguese), with

the following responses: makes products seem less healthy (n = 3), makes products seem more harmful to health (n = 1) and attracts the most attention (n = 2).

During the collective discussion, the message "ATTENTION: CONTAINS SWEETENER" (“*ATENÇÃO: CONTÉM EDULCORANTE*”, in Portuguese) was selected as the most appropriate. The shift in experts' opinion was attributed to several concerns: how Brazilian consumers would interpret a lengthy  message, the potential for the message’s size to either draw or deter consumer attention in the packaging context, and whether a non-recommendation statement could broaden or narrow the target audience of the warning.

Given the interconnected structure of the sessions, the second session presented two NSS warning label variations, both using the message "ATTENTION: CONTAINS NON-SUGAR SWEETENERS" (“*ATENÇÃO: CONTÉM EDULCORANTE*”, in Portuguese). The difference between the proposals was in typographic emphasis:  one version had the entire sentence in bold, while the other emphasized in bold only the word "attention" (“*atenção*”, in Portuguese), as illustrated in Figure 2. No consensus was reached in this session, so both versions were carried forward to the next one..

In the third session, the same two warning proposals were displayed in the context of packaging (Figure 3). Based on both individual questionnaire (Table 1) and the collective discussion, the position next to the FoPNL was considered the most appropriate.

**Table 1. Ranking of NSS warning positions on labels**

| | **Message in bold type (n = 8)** | | | **Word “attention” in bold type (n = 9)** | | |
|---|---|---|---|---|---|---|
| **Visibility** | **1º** | **2º** | **3º** | **1º** | **2º** | **3º** |

| | | | | | | |
|---|---|---|---|---|---|---|
| Next to the FoPNL | 6 | 2 | 0 | 7 | 2 | 0 |
| After the list of ingredients | 1 | 4 | 3 | 1 | 3 | 5 |
| Next to the sales denomination | 1 | 2 | 5 | 1 | 4 | 4 |
| **Attention** | | | | | | |
| Next to the FoPNL | 6 | 2 | 0 | 7 | 2 | 0 |
| After the list of ingredients | 1 | 2 | 5 | 1 | 2 | 6 |
| Next to the sales denomination | 1 | 4 | 3 | 1 | 5 | 3 |

FoPNL: front-of-package nutrition labeling.

Considering the content analysis of the collective discussion, the most frequently referenced codes were: attributes of NSS warning labels (n = 26), the attribute of the word "attention" attracting attention (n = 17), use of the word “attention” as a signal (n = 11), and the notion of non-recommendation for NSS consumption (n = 11). The most frequent codes were related to the specific characteristics of the warning label proposals, the design elements and the message of non-recommendation. Conversely, the least frequently referenced codes were: perception of healthiness and/or health risk (n = 7), inference about consumers opinions (n = 9), and expert's personal preference/opinion (n = 9), as shown in Table 2. The limited number of references about perception of healthiness and/or health risk is noteworthy, given that is one of the technical attributes experts were asked to evaluate and considering that seven of the participants were health professionals.

**Table 2. Codes, subcodes, description and number of references that emerged through the content analysis of the panel of experts.**

| Code | Subcode | Description | References |
|---|---|---|---|
| Attributes about NSS warning labels | - | Experts' statements regarding the attributes present within the labeling models (message, types of | 26 |

| | | | |
|---|---|---|---|
| | | presentation, alert size etc.) proposed to indicate the presence of NSS and on packaged foods and beverages. | |
| Attention | The attribute of attracting attention | Experts' statements regarding the NSS warning labels attract attention when they are seen. | 17 |
| | Use of the word attention as a sign | Experts' statements regarding the use of the term "attention" in the NSS warning labels. | 11 |
| Non-recommendation to consume NSS | - | Experts' statements with arguments not recommending the consumption of NSS for different audiences, contemplating scientific evidence arguments. | 11 |
| Inference about consumers opinions | - | Experts' statements with inferences about the possible opinion and/or interpretation of consumers regarding the labeling models for NSS in packaged foods and beverages. | 9 |
| Expert's personal preference/opinion | - | Experts' statements indicating their personal and/or professional preference/opinion on the NSS warning labels, considering possible mentions or disagreements regarding the questions asked in the panel dynamics. | 9 |
| Perception of healthiness and/or health risk | - | Experts' statements regarding their perception of healthiness and/or health risk in relation to the NSS warning labels. | 7 |

NSS: non-sugar sweetener.

## The NSS warning labels design

The size of the message was a point of discussion, as experts expressed a preference for shorter messages, that are quicker to read and more consistent with

other label elements already present on Brazilian packaging (such as information on gluten and lactose). Regarding the visual emphasis, there was a clear preference for presenting the entire message in bold, especially given the most appropriate placement for visibility and attention, according to the experts, was near the FoPNL, which is also in bold, or after the list of ingredients.

"*what would be, maybe it would more visible or be more informative, it would be a longer message, on attention and such for children and weight loss, but I keep thinking in terms of visual space on these packages, on these labels, that a very long text will need to use very small print and then it won't draw attention"* (woman, social communication experts).

## Attention (attracting attention and the use of a signal word)

Considering that some of the NSS warning labels adopted the signal word "attention", and that this was a specific attribute to be evaluated by experts, the attention code was subdivided into two subcategories: "the attribute of drawing attention" and "use of the word attention as a signal". For the first subcategory, participants associated the ability to attract attention with multiple elements, such as: the use of specific terms ("attention" and "not recommended"), bold formating, proportionality of the message, and label positioning. As for to the use of the word "attention" as a signal, its placement at the beginning of the message was seen as a strategy that could trigger awareness and convey a sense of alert.

During the second session, when readability and attention of two NSS warning models were analyzed individually, most experts agreed via questionnaire that

displaying the entire message in bold improved readability (n = 7) and increased attention (n = 8).

*"(...) the criterion was on getting people's attention, right? Showing that something can be, can present a risk. I think the word 'ATTENTION' makes a difference. I, we have heard this from some people and particularly from me too, if I see the word 'ATTENTION', that already turns on a red light in our head. So that was the first point, having 'ATTENTION' at the front."* (woman, nutrition expert).

Regarding design attributes, the use of the word "attention" at the beginning of the message and bold formatting were considered differentials that helped to emphasize the message and triggered a sense of alertness among the experts, which they believe could extend to Brazilian consumers. In addition, placing the NSS warning label next to the FoPNL was considered a strategic choice to improve consumer visibility and understanding, even for those unfamiliar with the term NSS.

## The inclusion of a non-recommendation sentence

Some proposed warning labels included a sentence recommending against NSS consumption by children and/or people attempting to control body weight. Experts noted that this phrasing could either broaden or restrict the perceived target audience. For instance, some consumers might assume that if a product is not suitable for children, it might also be unsuitable for adults, while others might ignore the warning if they are not part of the specified groups. This raised concern among experts, as NSS are not recommended for other groups as well.

*"If I'm not worried about weight control,and I'm not a child, I would consider, right, that this product would be recommended for me, (...) and it's not. So I would also like to know, as a healthy adult, I would like to know that that product is not meant for me"* (man, pharmacy expert).

It is worth highlighting that the statements about the non-recommendation message included healthiness arguments, since the absence of consumption recommendation is related to a potential health risk or benefit.

## Inference about consumers' opinions

Although the panel was designed to gather technical input, some responses reflected assumptions about consumers. Experts believed that consumers are less likely to read long sentences and are more impacted by short messages. The use of audience-specific phrases like "children" or "weight control" generated mixed reactions, because they could both restrict (disregard those who were not mentioned) or expand (understanding that, as it is not adequate for children, it is not suitable for anyone) the target audience. Finally, the main aspect highlighted about this topic was that the specific term for NSS in Portuguese (*edulcorante*) is not understood by the Brazilian population.

*"(...) I stopped at the NSS, I noted 'ATTENTION: CONTAINS NON-SUGAR SWEETENER'. I was very uncertain, but what made me select this option was because the feeling I have is that when we specify the audience, it can exclude me from the audience for whom it is harmful. So, I can think that if this is not suitable for children and those who are controlling their weight, then maybe this is not suitable for me, a person who is not a child and who is not controlling weight (...)"* (woman, psychology expert).

## Expert's personal preference

Some experts noted a discrepancy between their personal preferences and the answers they submitted, as the structured format of the questionnaires did not provide room for open-ended opinion sharing.

*“What I missed when I saw the block of questions was “what is your opinion on this? (...)”* (woman, nutrition expert).

## Perception of healthiness and/or health risk

Regarding those attributes, the experts used elements of the NSS warning labels for discussion. The term “attention” and the use of the non-recommendation phrase were elements indicated as important to interfere in the perception of the healthiness of products, especially the non-recommendation message that covers two audiences, children and people implicated in controlling body weight. In relation to health risk, only the non-recommendation for children was considered impactful. The use of too many terms on labels was mentioned as a possibility that led to a reductive view of products. Additionally, products without such warnings could be perceived as healthier, which is not always accurate. While the intention of warning labels is to inform, some experts noted that shaping consumer perceptions about health risks could be seen as exceeding that function.

*“The criterion was the question of getting people’s attention, right? To show what something can be, to present a risk. I think the word ‘ATTENTION’ makes a difference. I, we have heard this from some people and particularly from me too, if I see the word ‘ATTENTION’, that already lights up a red light in our head. So that was the first criterion, having the word ‘ATTENTION’ at the front. And the other was: It is not recommended for both children and weight control. So, among the options we had, it would be, let's say, a more complete sentence from the point of view of the less healthy, right?”* (woman, nutrition expert).

# Discussion

This study identified technical and personal opinions of experts from different areas of knowledge regarding specific attributes of proposed NSS warning labels. In Brazil, information about NSS is limited to the list of ingredients. However, studies on FoPNWL have shown that experts consider such information useful for guiding food

choices (47).While the structure nature of the questionnaire focused on specific attributes, some experts expressed a desire to share broader personal perspectives.

There are several limitations to the study. Conducting the expert's panel virtually may have hindered the ability to fully capture non-verbal communication. Additionally, despite efforts to diversify the group, it was challenging to recruit experts from different fields across the country with specific expertise on the topic. Nonetheless, purposive sampling enabled the inclusion of a diverse panel of professionals based in various Brazilian cities.

In Brazil, on food labeling regulation is coordinated by Anvisa, the national agency responsible for regulating overseeing and inspecting products and services that pose potential risks to public health. The regulatory process includes the contributions from government, academia, civil society organizations and industry. The most recent review of nutrition labeling regulations, which lasted six years, heavily relied on scientific evidence (41,42). Despite this, a specifc warning for NSS was not adopted with Anvisa stating that NSS labeling would be defined in the MERCOSUR agenda (48). Currently, Anvisa is leading a new regulatory agenda to revise NSS labeling and use. In 2024, the agency published a regulatory discussion paper and held technical meetings with stakeholders, including the food industry, academia and civil society.

Anvisa's discussion paper highlights the potential for improvement of NSS labeling and proposes the inclusion of a front-of-package warning, particularly addressing contraindications for children (49). The results of this study will be presented in due course within the ongoing regulatory process, with the aim of informing decisions related to public health and the right to information.

The use of food additives in products has been increasingly studied in the last decade. A study assessing the list of ingredients of foods and beverages available on the Brazilian market identified that of 9,856 products, 20.6% contain no food additives, 11.6% contained one additive, 19.8% contained between two and three, 23.2% contained between four and five and 24.8% six or more. The study also identified patterns of food additives combinations, with the fifth pattern being exclusively represented by NSS, which was associated with the following product categories: sugar and other non-caloric sweeteners, fruit-flavored drinks and other beverages. Currently, the most frequent food additives category in products sold in Brazil is flavoring(50). This highlights the importance of improving information about NSS, particularly since such details are currently confined to the ingredients list, which, despite being regulated, pose comprehension barriers for many consumers (24).

Several factors support the call for more transparent labeling of NSS in Brazil. These include the Chilean experience, where the implementation of FoPNL led to the total or partial replacement of added sugars for NSS (39,40); increasing evidence on the potential health risks associated with NSS consumption (13); the WHO`s conditional recommendation regarding NSS intake (23); and the absence of any specific NSS warning in the current Brazilian FoPNL system (41,42).

In contrast to regulated information, advertising claims on packaging often receive greater visual emphasis. Evidence shows that visual elements on labels interfere in the perception of products' healthiness(51,52), while FoPNL implementation has been linked to decreased perception of healthiness. To date, however, there are no studies that evaluated Brazilian consumers' perception of NSS warning labels.

One study involving 448 Brazilian consumers found that FoPNL reduced the perceived healthfulness of certain packaged products (e.g., chocolate milk with granola, mate tea with cookies). However, familiar brands and textual nutritional marketing claims also have significant effect on the perception of healthiness(47). This demonstrates that while FoPNL helps consumers identify highlighted nutrients, marketing strategies still shape perceived healthfulness.

In Mexico, studies have found that NSS warning labels reduce parents perceptions of a product healthfulness more effectively than excess sugar warning alone (53), and the presence of NSS warning influences the healthiness perception of beverages for children(54). Such findings support the potential role of NSS-specific warning labels in improving consumer understanding of product nutrition content. It is important to highlight that the presence of NSS in a product characterizes it as an UPFP, a group consistently associated with negative health outcomes ((5–11).

While evidence on NSS-specific labeling remains limited, data from Mexico suggest that such labels can reduce the perceived healthiness of UPFPs. Given the likelihood that UPFPs in Brazil may be reformulated by replacing added sugars with NSS following FoPNL implementation, it is crucial to ensure that consumers are provided with clear, accessible information.

# Conclusion

The study provides interdisciplinary evidence and expert insight, to support the development of NSS warning labels in Brazil. Considering the individual questionnaire and the content analysis of collective discussion, the experts' decision about the most appropriate NSS warning label was: "attention: contains non-sugar sweetener - not recommended to children and for weight control" (Atenção: contém edulcorante - não recomendado para crianças e para controle de peso, in Portuguese) and "attention:

contains non-sugar sweetener” (Atenção: contém edulcorante, in Portuguese). Experst recommended that these messages be displayed in bold and positioned on the main panel, near the FoPNL. This represents the first phase of the study and will guide the next steps in the validation of models among Brazilian consumers.

**Acknowledgments**

To all the experts who voluntarily participated in the panel and shared their knowledge of this study.

# References

1. Brazil. Ministry of Health. Brazilian National Health Surveillance Agency (Anvisa). INSTRUÇÃO NORMATIVA - IN N° 211, DE 1° DE MARÇO DE 2023 [Internet]. Brasília; 2023 mar. Disponível em: https://www.in.gov.br/en/web/dou/-/instrucao-normativa-in-n-211-de-1-de-marco-de-2023-468509746
2. De La Peña C. Artificial sweetener as a historical window to culturally situated health. Ann N Y Acad Sci [Internet]. 2010 [citado 1º de abril de 2025];1190:159–65. Disponível em: https://pubmed.ncbi.nlm.nih.gov/20388147/
3. Monteiro CA, Cannon G, Levy RB, Moubarac JC, Louzada MLC, Rauber F, et al. Ultra-processed foods: what they are and how to identify them. Public Health Nutr [Internet]. 1º de abril de 2019 [citado 14 de abril de 2025];22(5):936–41. Disponível em: https://pubmed.ncbi.nlm.nih.gov/30744710/
4. Monteiro CA, Cannon G, Levy R, Moubarac JC, Jaime P, Paula Martins A, et al. NOVA. The star shines bright. World Nutrition [Internet]. 7 de janeiro de 2016 [citado 14 de abril de 2025];7(1–3):28–38. Disponível em: https://worldnutritionjournal.org/index.php/wn/article/view/5
5. Lane MM, Gamage E, Du S, Ashtree DN, McGuinness AJ, Gauci S, et al. Ultra-processed food exposure and adverse health outcomes: umbrella review of epidemiological meta-analyses. BMJ [Internet]. 2024 [citado 14 de abril de 2025];384. Disponível em: https://pubmed.ncbi.nlm.nih.gov/38418082/
6. Nilson EAF, Ferrari G, Louzada MLC, Levy RB, Monteiro CA, Rezende LFM. Premature Deaths Attributable to the Consumption of Ultraprocessed Foods in Brazil. Am J Prev Med [Internet]. 1º de janeiro de 2023 [citado 14 de abril de 2025];64(1):129–36. Disponível em: https://pubmed.ncbi.nlm.nih.gov/36528353/
7. Taneri PE, Wehrli F, Roa-Díaz ZM, Itodo OA, Salvador D, Raeisi-Dehkordi H, et al. Association Between Ultra-Processed Food Intake and All-Cause Mortality: A Systematic Review and Meta-Analysis. Am J Epidemiol [Internet]. 1º de julho de 2022

[citado 14 de abril de 2025];191(7):1323–35. Disponível em: https://pubmed.ncbi.nlm.nih.gov/35231930/

8. Pagliai G, Dinu M, Madarena MP, Bonaccio M, Iacoviello L, Sofi F. Consumption of ultra-processed foods and health status: a systematic review and meta-analysis. Br J Nutr [Internet]. 14 de fevereiro de 2021 [citado 14 de abril de 2025];125(3):308–18. Disponível em: https://pubmed.ncbi.nlm.nih.gov/32792031/
9. De Amicis R, Mambrini SP, Pellizzari M, Foppiani A, Bertoli S, Battezzati A, et al. Ultra-processed foods and obesity and adiposity parameters among children and adolescents: a systematic review. Eur J Nutr [Internet]. 1º de agosto de 2022 [citado 14 de abril de 2025];61(5):2297–311. Disponível em: https://pubmed.ncbi.nlm.nih.gov/35322333/
10. Lane MM, Davis JA, Beattie S, Gómez-Donoso C, Loughman A, O'Neil A, et al. Ultraprocessed food and chronic noncommunicable diseases: A systematic review and meta-analysis of 43 observational studies. Obes Rev [Internet]. 1º de março de 2021 [citado 14 de abril de 2025];22(3). Disponível em: https://pubmed.ncbi.nlm.nih.gov/33167080/
11. Askari M, Heshmati J, Shahinfar H, Tripathi N, Daneshzad E. Ultra-processed food and the risk of overweight and obesity: a systematic review and meta-analysis of observational studies. Int J Obes (Lond) [Internet]. 1º de outubro de 2020 [citado 14 de abril de 2025];44(10):2080–91. Disponível em: https://pubmed.ncbi.nlm.nih.gov/32796919/
12. Brazil. Ministry of Health. Biblioteca Virtual em Saúde do Ministério da Saúde [Internet]. 2º ed. Brasília; 2014. 1–158 p. Disponível em: www.saude.gov.br/bvs
13. Organization WH, Rios-Leyvraz M, Montez J. Health effects of the use of non-sugar sweeteners: a systematic review and meta-analysis. 2022 [citado 14 de abril de 2025]; Disponível em: https://iris.who.int/handle/10665/353064
14. Shum B, Georgia S. The Effects of Non-Nutritive Sweetener Consumption in the Pediatric Populations: What We Know, What We Don't, and What We Need to Learn. Front Endocrinol (Lausanne) [Internet]. 1º de abril de 2021 [citado 14 de abril de 2025];12. Disponível em: https://pubmed.ncbi.nlm.nih.gov/33868167/
15. Suez J, Cohen Y, Valdés-Mas R, Mor U, Dori-Bachash M, Federici S, et al. Personalized microbiome-driven effects of non-nutritive sweeteners on human glucose tolerance. Cell [Internet]. 1º de setembro de 2022 [citado 14 de abril de 2025];185(18):3307-3328.e19. Disponível em: https://pubmed.ncbi.nlm.nih.gov/35987213/
16. Suez J, Korem T, Zeevi D, Zilberman-Schapira G, Thaiss CA, Maza O, et al. Artificial sweeteners induce glucose intolerance by altering the gut microbiota. Nature [Internet]. 9 de outubro de 2014 [citado 14 de abril de 2025];514(7521):181–6. Disponível em: https://pubmed.ncbi.nlm.nih.gov/25231862/
17. Lin J, Curhan GC. Associations of sugar and artificially sweetened soda with albuminuria and kidney function decline in women. Clin J Am Soc Nephrol [Internet]. 1º de janeiro de 2011 [citado 14 de abril de 2025];6(1):160–6. Disponível em: https://pubmed.ncbi.nlm.nih.gov/20884773/
18. Debras C, Chazelas E, Srour B, Druesne-Pecollo N, Esseddik Y, de Edelenyi FS, et al. Artificial sweeteners and cancer risk: Results from the NutriNet-Santé population-based cohort study. PLoS Med [Internet]. 1º de março de 2022 [citado 14 de abril de 2025];19(3). Disponível em: https://pubmed.ncbi.nlm.nih.gov/35324894/

19. Debras C, Chazelas E, Sellem L, Porcher R, Druesne-Pecollo N, Esseddik Y, et al. Artificial sweeteners and risk of cardiovascular diseases: results from the prospective NutriNet-Santé cohort. The BMJ [Internet]. 2022 [citado 14 de abril de 2025];378:e071204. Disponível em: https://pmc.ncbi.nlm.nih.gov/articles/PMC9449855/
20. Pearlman M, Obert J, Casey L. The Association Between Artificial Sweeteners and Obesity. Curr Gastroenterol Rep [Internet]. 1º de dezembro de 2017 [citado 14 de abril de 2025];19(12). Disponível em: https://pubmed.ncbi.nlm.nih.gov/29159583/
21. Fowler SPG. Low-calorie sweetener use and energy balance: Results from experimental studies in animals, and large-scale prospective studies in humans. Physiol Behav [Internet]. 1º de outubro de 2016 [citado 14 de abril de 2025];164(Pt B):517–23. Disponível em: https://pubmed.ncbi.nlm.nih.gov/27129676/
22. Chia CW, Shardell M, Tanaka T, Liu DD, Gravenstein KS, Simonsick EM, et al. Chronic Low-Calorie Sweetener Use and Risk of Abdominal Obesity among Older Adults: A Cohort Study. PLoS One [Internet]. 1º de novembro de 2016 [citado 14 de abril de 2025];11(11). Disponível em: https://pubmed.ncbi.nlm.nih.gov/27880832/
23. World Health Organization. Use of non-sugar sweeteners. WHO guideline. 2023;
24. de Carvalho TEM, Waisenberg A, Sato P de M, Mais LA, Martins APB, Jaime PC, et al. Consumer perceptions of non-caloric sweeteners and the content of caloric and non-caloric sweeteners in ultra-processed products in Brazil. Cien Saude Colet [Internet]. 2022 [citado 14 de abril de 2025];27(5):1989–2000. Disponível em: https://pubmed.ncbi.nlm.nih.gov/35544825/
25. Mexico. Ministry of Economy. Secretaría de Salud. REDUCCIÓN DEL COSTO DE CUMPLIMIENTO DE LAS NUEVAS OBLIGACIONES DE LA PROPUESTA REGULATORIA [Internet]. 2020 mar. Disponível em: https://www.dof.gob.mx/normasOficiales/8150/seeco11_C/seeco11_C.html
26. Argentina. National Executive Power. Lei_Argentina.gob.ar. 23 de março de 2022;1–46.
27. Organization PAH. Front-of-Package Labeling as a Policy Tool for the Prevention of Noncommunicable Diseases in the Americas. 2020 [citado 14 de abril de 2025]; Disponível em: https://iris.paho.org/handle/10665.2/52740
28. Ares G, Antúnez L, Curutchet MR, Giménez A. Warning labels as a policy tool to encourage healthier eating habits. Curr Opin Food Sci [Internet]. 1º de junho de 2023 [citado 14 de abril de 2025];51. Disponível em: https://www.researchgate.net/publication/371208898_Warning_labels_as_a_policy_tool_to_encourage_healthier_eating_habits
29. de Morais Sato P, Mais LA, Khandpur N, Ulian MD, Bortoletto Martins AP, Garcia MT, et al. Consumers' opinions on warning labels on food packages: A qualitative study in Brazil. PLoS One [Internet]. 1º de agosto de 2019 [citado 14 de abril de 2025];14(6). Disponível em: https://pubmed.ncbi.nlm.nih.gov/31242235/
30. Mediano Stoltze F, Busey E, Taillie LPS, Dillman Carpentier FR. Impact of warning labels on reducing health halo effects of nutrient content claims on breakfast cereal packages: A mixed-measures experiment. Appetite [Internet]. 1º de agosto de 2021 [citado 14 de abril de 2025];163. Disponível em: https://pubmed.ncbi.nlm.nih.gov/33789168/
31. Wogalter MS, & MCB. Warning design. In Information Design . Routledge, organizador. 2017. 347–364 p.

32. Rayner M, Vandevijvere S. INFORMAS Protocol: Food Labelling Module. 6 de dezembro de 2017 [citado 13 de maio de 2025]; Disponível em: /articles/journal_contribution/INFORMAS_Protocol_Food_Labelling_Module/5673643/1
33. Rojas CFU, Spinillo CG. Warning assessment: Information design contributions to the evaluation of communication efficacy of front of packaging nutrition labelling. InfoDesign [Internet]. 18 de junho de 2021 [citado 13 de maio de 2025];18(1):1–28. Disponível em: https://infodesign.org.br/infodesign/article/view/877
34. Spence C. On the psychological impact of food colour. Flavour 2015 4:1 [Internet]. 22 de abril de 2015 [citado 13 de maio de 2025];4(1):1–16. Disponível em: https://flavourjournal.biomedcentral.com/articles/10.1186/s13411-015-0031-3
35. Singh S. Impact of color on marketing. Management Decision [Internet]. 2006 [citado 13 de maio de 2025];44(6):783–9. Disponível em: https://www.researchgate.net/publication/235320162_Impact_of_color_on_marketing
36. Legge GE, Parish DH, Luebker A, Wurm LH. Psychophysics of reading XI Comparing color contrast and luminance contrast. Journal of the Optical Society of America A [Internet]. 1º de outubro de 1990 [citado 13 de maio de 2025];7(10):2002. Disponível em: https://pubmed.ncbi.nlm.nih.gov/2231110/
37. Crosbie E, Gomes FS, Olvera J, Rincón-Gallardo Patiño S, Hoeper S, Carriedo A. A policy study on front–of–pack nutrition labeling in the Americas: emerging developments and outcomes. The Lancet Regional Health - Americas [Internet]. 1º de fevereiro de 2023 [citado 13 de maio de 2025];18. Disponível em: https://pubmed.ncbi.nlm.nih.gov/36844016/
38. Chile. Ministerio de Salud. Ley 20606 de 06 de julio de 2012. Sobre composición nutricional de los alimentos y su publicidad. Ley 20.606 de 06 de julio de 2012. Sobre composición nutricional de los alimentos y su publicidad. Chile; 2012.
39. Rebolledo N, Reyes M, Popkin BM, Adair L, Avery CL, Corvalán C, et al. Changes in nonnutritive sweetener intake in a cohort of preschoolers after the implementation of Chile's Law of Food Labelling and Advertising. Pediatr Obes [Internet]. 1º de julho de 2022 [citado 14 de abril de 2025];17(7). Disponível em: https://pubmed.ncbi.nlm.nih.gov/35088571/
40. Zancheta Ricardo C, Corvalán C, Smith Taillie L, Quitral V, Reyes M. Changes in the Use of Non-nutritive Sweeteners in the Chilean Food and Beverage Supply After the Implementation of the Food Labeling and Advertising Law. Front Nutr [Internet]. 8 de novembro de 2021 [citado 14 de abril de 2025];8. Disponível em: https://pubmed.ncbi.nlm.nih.gov/34859036/
41. Brazil. Ministry of Health. Brazilian National Health Surveillance Agency (Anvisa). INSTRUÇÃO NORMATIVA-IN Nº 75, DE 8 DE OUTUBRO DE 2020 [Internet]. Brasília; 2020 out. Disponível em: https://www.in.gov.br/en/web/dou/-/instrucao-normativa-in-n-75-de-8-de-outubro-de-2020-282071143
42. Brazil. Ministry of Health. Brazilian National Health Surveillance Agency (Anvisa). RESOLUÇÃO DE DIRETORIA COLEGIADA - RDC Nº 429, DE 8 DE OUTUBRO DE 2020 [Internet]. Brasília; 2020 out. Disponível em: https://www.in.gov.br/en/web/dou/-/resolucao-de-diretoria-colegiada-rdc-n-429-de-8-de-outubro-de-2020-282070599
43. POPPER R. How are foresight methods selected? Foresight. 2008;10(6):62–89.
44. Orientador G. Painel de Especialistas e Delphi: Métodos complementares na elaboração de estudos de futuro. 2018; Disponível em: www.embrapa.br/fale-conosco/sac

45. Rodrigues RM, Souza ADM, Bezerra IN, Pereira RA, Yokoo EM, Sichieri R. Most consumed foods in Brazil: evolution between 2008-2009 and 2017-2018. Rev Saude Publica [Internet]. 2021 [citado 13 de maio de 2025];55(Supl 1). Disponível em: https://pubmed.ncbi.nlm.nih.gov/34852162/
46. Minayo M. O desafio do conhecimento: pesquisa qualitativa em saúde. Hucitec. 2006;(9):406.
47. França CG, de Alcantara M, Deliza R. Effect of nutrition warning, brand, and claims on the perception of product healthiness by Brazilian consumers. Food Res Int [Internet]. 1º de fevereiro de 2025 [citado 14 de abril de 2025];201. Disponível em: https://pubmed.ncbi.nlm.nih.gov/39849690/
48. Mais LA, Borges CA, Khandpur N, Duran AC, Martins APB. Brazil's nutrition labeling regulation: Challenges ahead on the path to guaranteeing consumer's right to adequate information. Front Nutr [Internet]. 22 de novembro de 2022 [citado 14 de abril de 2025];9. Disponível em: https://pubmed.ncbi.nlm.nih.gov/36483931/
49. Agência Nacional de Vigilância Sanitária (Anvisa). Uso de Edulcorantes em Alimentos: Documento para discussão regulatória. 2024;1–105.
50. Montera VDSP, Martins APB, Borges CA, Canella DS. Distribution and patterns of use of food additives in foods and beverages available in Brazilian supermarkets. Food Funct [Internet]. 31 de agosto de 2021 [citado 14 de abril de 2025];12(17):7699–708. Disponível em: https://pubs.rsc.org/en/content/articlehtml/2021/fo/d1fo00429h
51. Techawachirakul M, Pathak A, Calvert GA. That sounds healthy! Audio and visual frequency differences in brand sound logos modify the perception of food healthfulness. Food Qual Prefer. 1º de julho de 2022;99.
52. Hallez L, Vansteenbeeck H, Boen F, Smits T. Persuasive packaging? The impact of packaging color and claims on young consumers' perceptions of product healthiness, sustainability and tastiness. Appetite [Internet]. 1º de março de 2023 [citado 14 de abril de 2025];182. Disponível em: https://pubmed.ncbi.nlm.nih.gov/36567019/
53. Calderon C, Aburto TC, Batis C, Contreras-Manzano A, Barquera S. Influence of the cautionary legend on non-nutritive sweetener (NNS) on preference and healthfulness perception. PLoS One [Internet]. 1º de novembro de 2024 [citado 14 de abril de 2025];19(11). Disponível em: https://pubmed.ncbi.nlm.nih.gov/39585876/
54. Arellano-Gómez LP, Jáuregui A, Nieto C, Contreras-Manzano A, Quevedo KL, White CM, et al. Effects of front-of-package caffeine and sweetener disclaimers in Mexico: cross-sectional results from the 2020 International Food Policy Study. Public Health Nutr [Internet]. 2 de dezembro de 2023 [citado 14 de abril de 2025];26(12):3278–90. Disponível em: https://pubmed.ncbi.nlm.nih.gov/37781769/

# Supporting information

**S1 File. Panel of expert's guide**
**S2 File. Expert panel codebook**

Figure Click here to access/download;Figure;Fig 1.png

- CONTÉM EDULCORANTE
  (in English: CONTAINS NON-SUGAR SWEETENER)

- ATENÇÃO: CONTÉM EDULCORANTE
  (in English ATTENTION: CONTAINS NON-SUGAR SWEETENER)

- CONTÉM EDULCORANTE - NÃO RECOMENDADO PARA CRIANÇAS
  (in English CONTAINS NON-SUGAR SWEETENER - NOT RECOMMENDED FOR CHILDREN)

- ATENÇÃO: CONTÉM EDULCORANTE - NÃO RECOMENDADO PARA CRIANÇAS
  (in English ATTENTION: CONTAINS NON-SUGAR SWEETENER - NOT RECOMMENDED FOR CHILDREN)

- CONTÉM EDULCORANTE - NÃO RECOMENDADO PARA CONTROLE DE PESO
  (in English CONTAINS NON-SUGAR SWEETENER - NOT RECOMMENDED FOR WEIGHT CONTROL)

- ATENÇÃO: CONTÉM EDULCORANTE - NÃO RECOMENDADO PARA CONTROLE DE PESO
  (in English ATTENTION: CONTAINS NON-SUGAR SWEETENER: NOT RECOMMENDED FOR WEIGHT CONTROL)

- CONTÉM EDULCORANTE - NÃO RECOMENDADO PARA CRIANÇAS E PARA CONTROLE DE PESO
  (in English CONTAINS NON-SUGAR SWEETENER: NOT RECOMMENDED FOR CHILDREN AND FOR WEIGHT CONTROL)

Figure
Click here to access/download;Figure;Fig 2.png

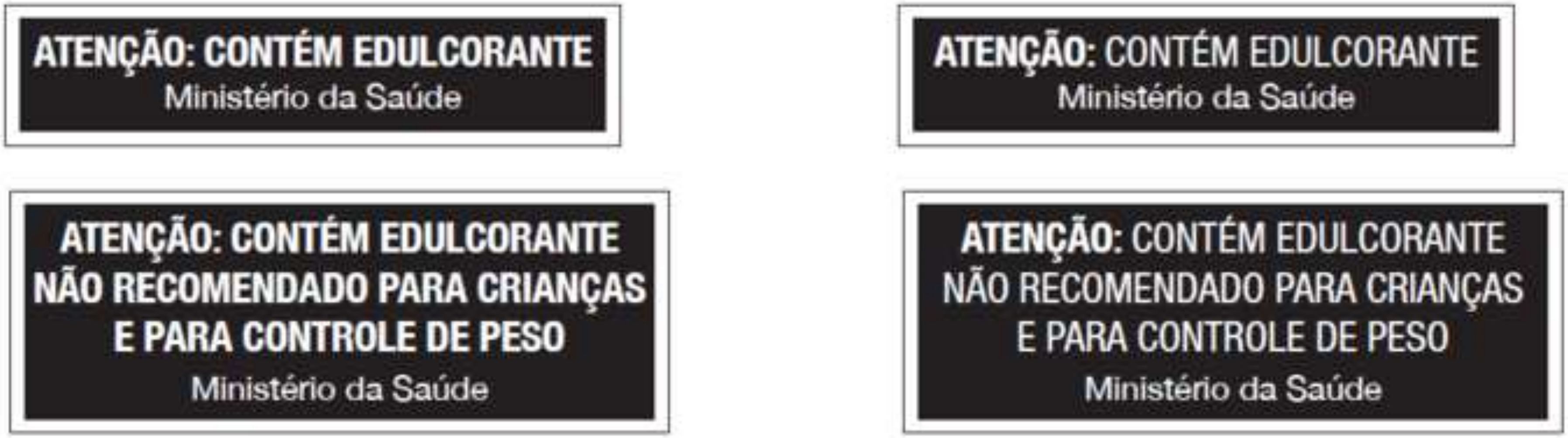

Figure

Click here to access/download;Figure;Fig 3.png

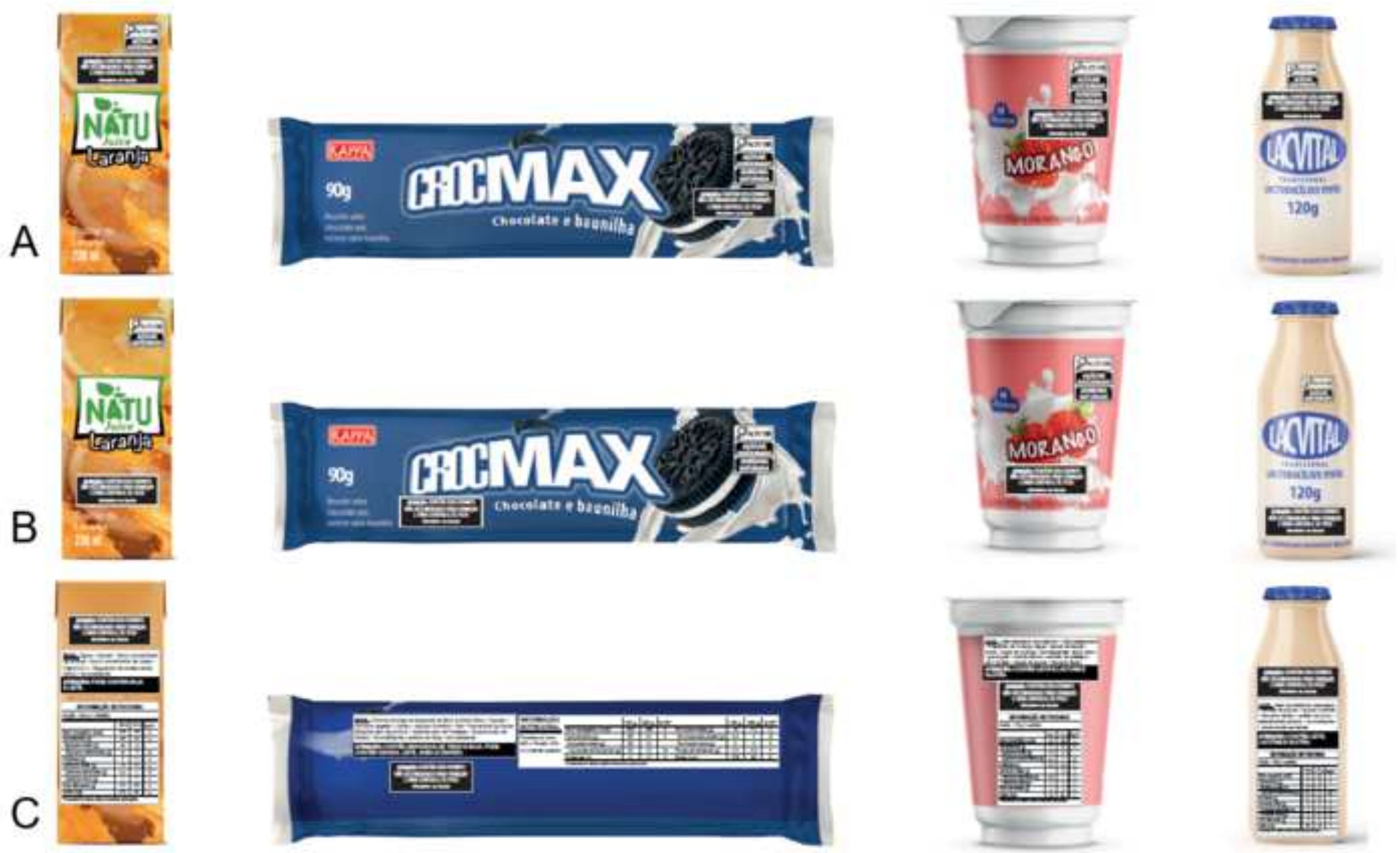

Click here to access/download
**Supporting Information**
S1_File.docx

Click here to access/download
**Supporting Information**
S2_File.docx
