# Supplementary material for: Experts contributions to the development of a non-sugar sweeteners warning label for Brazilian food products
Source: PLoS One. 2025 Sep 3;20(9):e0331302. doi: 10.1371/journal.pone.0331302 (PMC12407391; doi:10.1371/journal.pone.0331302)
Supplement: Attachment — Submitted filename: Response to Reviewers.pdf [file pone.0331302.s005.pdf]

Dear Editor and Reviewers,

Thank you for the opportunity to revise our manuscript entitled “Experts’ contributions to the development of a non-sugar sweeteners warning label for Brazilian food products” (PONE-D-25-27597). We have carefully considered all comments and suggestions, and we are pleased to submit a revised version for your consideration.

Our point-by-point responses to the reviewers’ and editors’ comments are detailed below in red. Revisions made to the manuscript are indicated using the “Track Changes” feature in the attached file. Additionally, we are submitting a clean version of the revised manuscript, without tracked changes, as requested.

We look forward to hearing from you regarding the outcome of the review.

Best regards,
The authors.

Required Revisions for Acceptance:

1. Abstract: Clearly highlight the main findings of the study, including the two preferred warning messages and their key design attributes.
We thank the reviewer for this valuable observation. We agree that the main findings were not clearly highlighted in the original abstract. We have revised the abstract accordingly to emphasize these results and improve this clarity.

2. Introduction:

- Improve the transition between the global health context and the Brazilian regulatory landscape by explicitly connecting WHO guidelines to the national policy gap earlier in the text.
Thank you for this valuable suggestion. We agree that the connection was not sufficiently clear. To address this, we have relocated the paragraphs to establish an earlier and more explicit link between the WHO guidelines and the Brazilian regulatory context.

- Revise phrasing for conciseness where applicable (e.g., “Recent scientific evidence has associated” → “Recent evidence associates”).
We appreciate your recommendation regarding phrasing conciseness. We have carefully revised the introduction, identified additional similar instances, and made the necessary adjustments to improve clarity and conciseness throughout the text.

3. Materials and Methods:

- Provide more detailed information on how experts were identified and selected (e.g., through professional networks, databases, or institutional affiliations) to ensure reproducibility.
Thank you for the suggestion. We acknowledge that this additional information was missing and have now included it in the Methods section, specifically under the sample description.

- Discuss how the lack of non-verbal communication in virtual panels may have impacted consensus-building.
We appreciate the observation. After consideration, we determined that it would be more appropriate to address the potential influence of the lack of non-verbal communication on consensus-building within the Discussion section, as it aligns with the broader reflection on study limitations. This placement ensures that the issue is contextualized alongside other limitations of the research design.

- Include examples of the questionnaire (e.g., Google Forms) as supplementary material to enhance transparency.
Thank you for the suggestion. The questions from all questionnaires, along with their corresponding instructions, have been compiled and included as a new supplementary file. Following the revision, this is now listed as Supplementary Material 2.

4. Results:

- Support expert assumptions about Brazilian consumers' understanding of the term "edulcorante" with references to existing literature on label comprehension, if available.
We thank you for noting the need to include this information. We have added a brief paragraph at the end of the inference about consumers' opinions results, supported by one specific study with Brazilian consumers.

- Expand on why health risk perception was less emphasized by professionals, considering the potential influence of design prioritization.
We thank you for underscoring this point. We included a paragraph offering a brief explanation of why health risk perception was less emphasized by the professionals, considering the potential influence of design prioritization. This paragraph was added after the presentation of the qualitative findings related to perception of healthiness and/or health risk.

- Include a brief comparison between Brazil's proposed warning labels and those implemented in Argentina and Mexico. Address whether the experts' preferences align with these regional approaches.
We thank you for this thoughtful suggestion. We incorporated this comparison in the final paragraph of the Results section.

5. Discussion:

- Discuss regulatory feasibility, including anticipated barriers such as industry resistance or consumer misinterpretation, and explain how the proposed labels may help to overcome them.
We appreciate the highlight for this important point. The discussion section has been revised to incorporate all these considerations.

- As the study is described as the "first phase," clearly outline the next steps, especially the planned consumer validation study, including its expected methodology and scope.

Thank you for highlighting the importance of including this information. We have revised the manuscript and this information, previously located in the Conclusion section, has been relocated to the Discussion with the additional details regarding the subsequent phases of the research.

- Eliminate redundant content, particularly repetitive references to international experiences (e.g., Chile's reformulation policies).
Thank you for your valuable suggestion. We reviewed and removed redundant elements and in addition, to improve the clarity of the manuscript, some specific information related to the results that were previously in the Discussion section have been relocated to the Results section.

6. References: Ensure that all references follow a consistent formatting style, as required by PLOS ONE.
Thank you for pointing this out. The references have been thoroughly reviewed and formatted according to the PLOS ONE guidelines.

Journal requirements:

When submitting your revision, we need you to address these additional requirements.

1. Please ensure that your manuscript meets PLOS ONE's style requirements, including those for file naming. The PLOS ONE style templates can be found at https://journals.plos.org/plosone/s/file?id=wjVg/PLOSOne_formatting_sample_main_body.pdf and https://journals.plos.org/plosone/s/file?id=ba62/PLOSOne_formatting_sample_title_authors_affiliations.pdf
We appreciate the observation. All supplementary material files have been appropriately renamed in accordance with PLOS ONE's file naming requirements, both within the submission system and in the documents themselves.

2. Please include your full ethics statement in the 'Methods' section of your manuscript file. In your statement, please include the full name of the IRB or ethics committee who approved or waived your study, as well as whether or not you obtained informed written or verbal consent. If consent was waived for your study, please include this information in your statement as well.
Thank you for highlighting this observation. The full ethics statement, as described in the submission form, has been included in the Methods section of the manuscript.

3. We note that the grant information you provided in the 'Funding Information' and 'Financial Disclosure' sections do not match.

When you resubmit, please ensure that you provide the correct grant numbers for the awards you received for your study in the 'Funding Information' section.
Thank you for bringing this to our attention. We would like to clarify that the authors did not receive any direct funding for this study. However, a resource was made available by the

Pan American Health Organization (PAHO), which was used specifically to cover the cost of transcription services for the focus group recordings. This payment was made directly to the company responsible for the transcription and was not managed by the authors. This information has been removed from the funding statement and a general acknowledgment to PAHO has been included in the manuscript instead.

5. Review Comments to the Author

Reviewer #1: The review is an attachment below completely

Abstract: The abstract could better highlight the study's key findings (e.g., the two preferred warning messages and their design attributes).

Introduction: The transition from global/NSS health risks to Brazil's labeling gap could be smoother. Consider adding a sentence explicitly linking WHO guidelines to Brazil's regulatory context earlier.

We thank you very much for your thorough and thoughtful review. We truly appreciate the attention dedicated to the corrections.

Reviewer #2: Conducting a qualitative study is quite difficult in almost all fields of science. It was carried out systematically and the method section was very well defined. I congratulate you on your unique work, prepared with utmost care.

We thank you very much for your thorough and thoughtful review. We truly appreciate the attention dedicated to the corrections and we are also especially grateful for your comment regarding the study design. It is very encouraging to receive such positive feedback on our study.
